# Supplementary figures and images for: Informing epidemic (research) responses in a timely fashion by knowledge management – a Zika virus use case
Source: Biol Open. 2020 Dec 2;9(12):bio053934. doi: 10.1242/bio.053934 (PMC7725600; doi:10.1242/bio.053934)

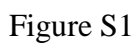

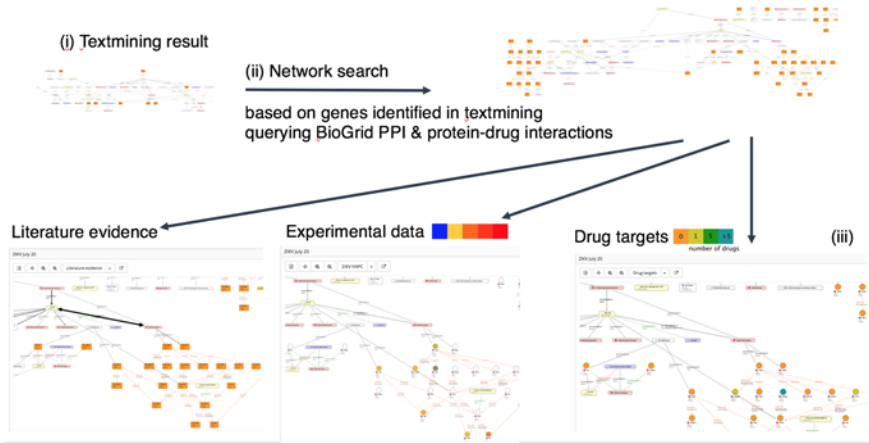

Figure S2

Supplement: Supplementary information [file biolopen-9-053934-s1.pdf]
